# PT320, Sustained-Release Exendin-4, Mitigates L-DOPA-Induced Dyskinesia in a Rat 6-Hydroxydopamine Model of Parkinson’s Disease

**DOI:** 10.3389/fnins.2020.00785

**Published:** 2020-08-11

**Authors:** Seong-Jin Yu, Shuchun Chen, Yung-Yung Yang, Elliot J. Glotfelty, Jin Jung, Hee Kyung Kim, Ho-Il Choi, Doo-Sup Choi, Barry J. Hoffer, Nigel H. Greig, Yun Wang

**Affiliations:** ^1^Center for Neuropsychiatric Research, National Health Research Institutes, Zhunan, Taiwan; ^2^Drug Design and Development Section, Translational Gerontology Branch, Intramural Research Program, National Institute on Aging, National Institutes of Health, Baltimore, MD, United States; ^3^Department of Neuroscience, Karolinska Institutet, Stockholm, Sweden; ^4^Peptron Inc., Daejeon, South Korea; ^5^Departments of Molecular Pharmacology and Experimental Therapeutics, Mayo Clinic College of Medicine and Science, Rochester, MN, United States; ^6^Department of Neurosurgery, Case Western Reserve University School of Medicine, Cleveland, OH, United States

**Keywords:** Parkinson’s disease, levodopa, L-DOPA-induced dyskinesia, glucagon-like peptide-1, exendin-4, PT320, PT302, exenatide

## Abstract

**Background:**

We previously demonstrated that subcutaneous administration of PT320, a sustained-release (SR) form of exendin-4, resulted in the long-term maintenance of steady-state exenatide (exendin-4) plasma and target levels in 6-hydroxydopamine (6-OHDA)-pretreated animals. Additionally, pre- or post-treatment with PT320 mitigated the early stage of 6-OHDA-induced dopaminergic neurodegeneration. The purpose of this study was to evaluate the effect of PT320 on L-3,4-dihydroxyphenylalanine (L-DOPA)-induced abnormal involuntary movements (AIMs) in the rat 6-OHDA model of Parkinson’s disease.

**Methods:**

Adult male Sprague–Dawley rats were unilaterally lesioned in the right medial forebrain bundle by 6-OHDA. L-DOPA and benserazide were given daily for 22 days, starting from 4 weeks after lesioning. PT320 was co-administered weekly for 3 weeks. AIM was evaluated on days 1, 16, and 22 after initiating L-DOPA/benserazide + PT320 treatment. Brain tissues were subsequently collected for HPLC measurements of dopamine (DA) and metabolite concentrations.

**Results:**

L-DOPA/benserazide increased AIMs of limbs and axial as well as the sum of all dyskinesia scores (ALO) over 3 weeks. PT320 significantly reduced the AIM scores of limbs, orolingual, and ALO. Although PT320 did not alter DA levels in the lesioned striatum, PT320 significantly attenuated 6-OHDA-enhanced DA turnover.

**Conclusion:**

PT320 attenuates L-DOPA/benserazide-induced dyskinesia in a 6-OHDA rat model of PD and warrants clinical evaluation to mitigate Parkinson’s disease in humans.

## Introduction

Levodopa, also known as L-3,4-dihydroxyphenylalanine (L-DOPA), is the precursor of dopamine (DA) and is currently the most commonly used medication for Parkinson’s disease (PD). The use of L-DOPA elevates dopamine (DA) synthesis in the lesioned substantia nigra and restores motor functions in PD patients. However, chronic administration of L-DOPA is often associated with abnormal involuntary movements (AIMs), also called levodopa-induced dyskinesia (LID) in PD patients. Early clinical studies have shown that 20–50% of PD patients developed dyskinesia within 5 years after the initiation of L-DOPA treatment (Rascol et al., 2000; Manson et al., 2012; Bjornestad et al., 2016). The severity of dyskinesia positively correlates with disease duration, Hoehn–Yahr stage, and duration of L-DOPA treatment (Nicoletti et al., 2016). Other studies also suggest that the disease severity and dose of L-DOPA are more important than the duration of L-DOPA treatment for the development of LID (Nutt et al., 2010; Espay et al., 2018).

LID has also been established in experimental animals. Chronic administration of L-DOPA to unilaterally 6-OHDA-lesioned rats has been widely used to examine AIMs (Lundblad et al., 2002). Similar to the PD patients, LID in the lesioned rats significantly correlates with the dose of L-DOPA and the magnitude of DA depletion (Putterman et al., 2007).

**FIGURE 1:**
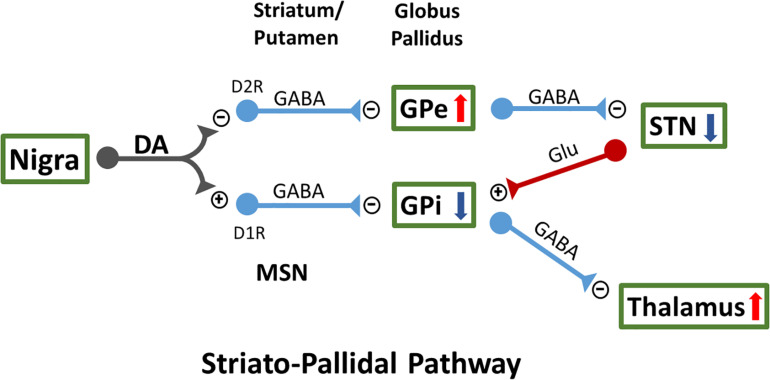
Two classical striatopallidal pathways of external segment of globus pallidus (GPe) and internal segment of globus (GPi) regulate neuronal activity in STN and thalamus as well as movement. MSN, medium spiny neurons; STN, subthalamic nucleus.

DA is a key neurotransmitter modulating normal movement. DA, released from the A9 neurons of the substantia nigra pars compacta (SNc) DA-ergic neurons, interacts with GABA-ergic medium spiny neurons (MSNs) within the dorsal striatum mainly comprised of caudate and putamen. There are two classical striatopallidal pathways (Figure 1). DA differentially inhibits the indirect GPe (external segment of globus pallidus) pathway through D2 receptors (D2R)-expressing MSNs, while it activates the direct GPi (internal segment of globus in primates or entopeduncular nucleus in rodents) pathway through D1R-expressing MSNs (Durieux et al., 2011; Gerfen and Surmeier, 2011). These interactions result in activation of GPe and suppression of neuronal activity in subthalamic nucleus (STN) and GPi, which further regulates thalamic neuronal activity and facilitates movement (Nambu et al., 2002). In pathological conditions, such as PD, reduction of dopaminergic innervation to caudate and putamen leads to overactivity of GABA-ergic inputs to GPe, which then suppresses the inhibitory outputs from the GPe to STN (Petri et al., 2013), activates STN and GPi neurons, and reduces neuronal firing in the thalamus. DA denervation also activates the GPi neurons through the direct striatopallidal pathway. Lesioning the STN or GPi induces marked functional improvement in 6-OHDA-lesioned rats (Touchon et al., 2004), 1-methyl-4-phenyl-1,2,3,6-tetrahydropyridine (MPTP) -treated monkeys (Bergman et al., 1990), and PD patients (Baron et al., 2000). On the other hand, L-DOPA or DA agonists can overstimulate DA receptors in the direct and indirect pathways in the lesioned brain, reduce neuronal firing in the STN and GPi while activating the thalamus, and result in increasing involuntary movements in MPTP-treated monkeys (Papa et al., 1999) and PD patients (Merello et al., 1999). Besides the interaction with the striatopallidal pathway, several other mechanisms have been suggested for LID (Jenner, 2008).

Since AIM is mainly induced after chronic administration of L-DOPA or DA-ergic agonists, agents that are non-DA-ergic molecules that possess less L-DOPA side effects are being increasingly studied for PD treatment (Fox et al., 2008). We and others previously demonstrated that the endogenous incretin glucagon-like peptide-1 (GLP-1) as well as exendin-4 (also known as exenatide), a long-acting GLP-1 receptor (GLP-1R) agonist approved for the treatment of type 2 diabetes mellitus (T2DM) (Drucker, 2018; Gentilella et al., 2019), protect tyrosine hydroxylase immunoreactivity (TH-IR) in primary ventromesenchephalic neurons from 6-OHDA lesioning. Infusion of exendin-4 into the lateral ventricle also mitigated the loss of TH-IR, preserved DA levels in the SNc, and improved the behavioral function of mice receiving MPTP (Li et al., 2009). Such GLP-1R-mediated protection has been broadly found across animal models of PD (Kim et al., 2017; Athauda and Foltynie, 2018; Holscher, 2020) as well as in other neurodegenerative disorders (Glotfelty et al., 2019). Importantly, clinical studies have demonstrated that PD patients taking exendin-4 for 1 year had better motor skills than those on placebo (Athauda et al., 2017, 2019). Besides its neuroprotective effects, exendin-4 has been reported to reduce LID in rats, with repeated administration of exendin-4 starting from the seventh day after 6-OHDA-lesioning resulting in lowering L-DOPA (10 mg/kg/day)-mediated AIM scores in 6-OHDA-lesioned rats (Abuirmeileh et al., 2012). These data suggest that activation of the GLP-1R can reduce the progression of DA degeneration and LID.

Major limitations of GLP-1R agonists, such as GLP-1, for clinical use are their relatively short half-life and, as peptide-based drugs, limited brain uptake (Glotfelty et al., 2020). A key amino acid change at the N-terminal of GLP-1 prevents breakdown by dipeptidyl peptidase-4 (DPP4) to extend its half-life from 1.5 min to 2.4 h for exendin-4 (Drucker, 2018). This results in the twice-daily clinical formulation *Byetta* that is considered the short-acting drug version. In contrast, the application of sustained-release (SR) technology to exendin-4 provides the capability to continuously release the same peptide present in *Byetta* over weeks to months after a single acute subcutaneous (s.c.) administration, resulting in the longer-term formulations PT320 (1 or 2 weeks) and *Bydureon* (1-week administration). Such technology provides the opportunity to optimize the beneficial potential of drug treatment for a chronic disorder by maintaining steady-state plasma levels as a source to maintain the brain target concentration (Li et al., 2019).

We recently reported that systemic administration of PT320 (also called PT302), SR exendin-4, given once every 2 weeks to unilaterally 6-OHDA-lesioned rats, provides sustained plasma exendin-4 levels (Chen et al., 2018). Pre- and post-treatment with PT320 significantly reduced methamphetamine-induced rotation and increased TH-IR in the lesioned SNc and striatum in these unilaterally 6-OHDA-lesioned rats. Furthermore, there was a significant correlation between exendin-4 plasma levels and TH-IR in the 6-OHDA-lesioned side SNc and striatum. These data suggest that PT320 provides long-lasting exendin-4 release and reduces dopaminergic neurodegeneration in this experimental model of PD. The use of PT320 in LID, however, has not been examined previously.

The purpose of this study was to evaluate the effect of PT320 on the L-DOPA/benserazide-mediated dyskinesia in a rat 6-OHDA model of Parkinsonism. Three doses of PT320 were administered over 3 weeks together with daily L-DOPA/benserazide (a peripherally acting aromatic L-amino acid decarboxylase inhibitor). We found that PT320 normalized DA turnover in the striatum and reduced LID behavior in these lesioned animals. Our data support the future clinical use of PT320 as a co-treatment with L-DOPA for PD.

## Materials and Methods

### Animals

Adult male Sprague–Dawley rats were used for this study. Experimental procedures followed the guidelines of the “Principles of Laboratory Care” (National Institutes of Health publication No. 86-23, 1996) and were approved by the Animal Care and Use Committee. Rats were fed with a regular chow diet and kept on a 12 h light/dark cycle at 25 ± 2°C. Animals were randomly assigned into four groups: Group 1 (sham operated); Group 2 (6-OHDA lesioned, no L-DOPA); Group 3 (lesioned + L-DOPA/benserazide + vehicle); and Group 4 (lesioned + L-DOPA/benserazide + PT320).

### PT320

PT320 (previously termed PT302) is an SR formulation of exendin-4 (exenatide). Powdered PT320 used in our study (Lot PT3025014) was of clinical-grade material, similar to that used in prior human studies (Gu et al., 2014), and contained a mixture of polymers (98%) and exendin-4 (2%). Specifically, exendin-4 was incorporated into poly(lactic-co-glycolic acid) (PLGA) microspheres of 20 μm diameter utilizing a proprietary ultrasonic spray drying process (SmartDepot^TM^, Peptron Inc, 2020) together with the use of an L-lysine coating to regulate the initial release burst of peptide (Li et al., 2019). The composition of the diluent used to prepare the PT320 suspension was 0.5% carboxymethylcellulose sodium, 5.0% D-mannitol, and 0.1% Tween 80 (pH 6.66) in sterile, double-distilled water as also used when PT320 was administered to humans. PT320 was freshly prepared in diluent within an hour of administration, maintained on wet ice (4°C), and thoroughly mixed (by vortex) immediately before each injection.

### 6-OHDA Lesioning and Drug Treatment

**FIGURE 2:**
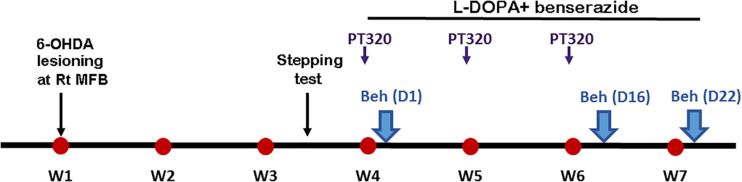
Timeline of drug treatment. Animals were screened by a stepping test at 3 weeks (W3) after unilateral 6-hydroxydopamine (6-OHDA) lesioning in the right medial forebrain bundle (Rt MFB). L-3,4-dihydroxyphenylalanine/benserazide was administered daily (i.p., L-DOPA: 6 mg/kg/day + benserazide 15 mg/kg/day) for 22 days. PT320 (100 mg/kg containing 2 mg/kg exendin-4) was administered subcutaneously once a week (W4, W5, and W6). Behavioral (Beh) evaluations of AIMs were examined on day 1 (D1), D16, and D22 after initiating L-DOPA/benserazide treatment (D0).

Thirty minutes prior to surgery, rats were given desipramine intraperitoneally (25 mg/kg; i.p.) to block noradrenergic uptake of 6-OHDA. Animals were anesthetized with 3% isoflurane. 6-OHDA (3 μg/μl × 2.5 μl dissolved in 0.1% ascorbic acid) was stereotactically injected into the right medial forebrain bundle (coordinates: −3.6 mm rostral and 1.6 mm lateral to bregma, 7.5 mm below the skull) at 0.25 μl/min over a 10 min period. Animals were allowed to recover for 3 weeks following the 6-OHDA lesion and then received drug treatment. Specifically, L-DOPA, dissolved in saline together with benserazide (15 mg/kg), was administered i.p. at a dose of 6 mg/kg/day for 22 days. PT320 (100 mg/kg, containing 2 mg/kg exendin-4 clinical-grade material) was administered subcutaneously once a week (three times in total) at 1 h before L-DOPA/benserazide administration. The timeline of the experiment is shown in Figure 2.

### Behavioral Tests

(1) A “Stepping test” was used to screen the success of lesioning at 3 weeks following the surgery. This was used rather than using methamphetamine-induced rotation to avoid baseline shifts due to sensitization. Briefly, the experimenter took the rat with one hand holding both hindlimbs and the other hand holding one of the forelimbs. The free paw was placed in contact with a flat surface. The experimenter then moved the animal slowly sideways in forward and backward directions. The number of adjusting steps taken by the rat was counted for both paws in the backward and forward direction. All animals displaying limb hypokinesia contralateral to the lesion were selected for subsequent study.
(2) L-dopa/benserazide-induced dyskinesia (LID) was examined by the abnormal involuntary movements (AIMs) test. Animals were placed in clear Perspex boxes (22 cm × 34 cm × 20 cm). Each rat was observed for 1 min at 30 min intervals following L-DOPA/benserazide administration over a 3 h period. Three subtypes of AIMs were assessed: (i) limb – random uncontrollable movements of forelimb contralateral to the lesion; (ii) orolingual – excess chewing and jaw movements with protrusion of the tongue; and (iii) axial – dystonic postures or choreiform twisting of the neck and upper body toward the contralateral side. The ALO score is the sum of all AIMs (axial, limb, and orolingual) scores. The severity of each AIM was scored non-parametrically between 1 and 4, based upon the following criteria:

1 = present for less than 30 s

2 = present for more than 30 s

3 = present throughout a minute but suppressed by external stimuli

4 = present throughout a minute but not suppressible by external stimuli

### HPLC Analysis and Electrochemical Detection

After final behavioral testing, animals were euthanized; their brains were quickly removed, placed in an ice-cold glass dish, and rapidly dissected on ice. Both lesioned and non-lesioned side striata were dissected, placed in an Eppendorf tube, frozen on dry ice, and stored at < −70°C before HPLC analysis. On the day of biochemical analysis, tissues were homogenized in 200 μl of 0.1 N perchloric acid (HClO_4_), sonicated, and centrifuged at 13,000 rpm for 30 min at 4°C. Aliquots (50 μl) of the supernatants were diluted in HCLO4 (1:4 v/v) before the injection into the HPLC system. The tissue concentrations of DA and metabolites were measured by HPLC coupled to the coulometric detection system. The mobile phase of the HPLC system was composed of methanol (7%), NaH_2_PO_4_ (70 mM), triethylamine (100 μl/L), EDTA (0.1 mM), and sodium octyl sulfate (100 mg/L) diluted in deionized water (pH 4.2, adjusted with orthophosphoric acid). It was filtered (0.22 μm) before its introduction in the system. The mobile phase was delivered through the HPLC column (Hypersyl, C18, 15 cm × 4.6 mm, particle size 5 μm) at a flow rate of 1.2 ml/min using an HPLC pump. The column was protected by a Brownlee–Newgard precolumn (RP-8, 15 × 3.2 mm, 7 μm.). The injection of the samples (10 μl) was carried out by a manual injection valve (Rheodyne, model 7725i) equipped with a loop of 20 μl. The compounds exited the column at different retention times and passed into the coulometric detection cell (Cell 5014, ESA) equipped with two electrodes. The potential of these two electrodes was fixed via the coulometric detector at + 350 mV (oxidation) and −270 m (reduction), respectively. The calibration curves were performed once the peaks in a standard solution (1 ng/10 μl) were well separated in the chromatogram. Calibration curves were performed using three concentrations of DA, DOPAC, and HVA injected three times each with an acceptable *r* = 0.99. Standard solutions were used before each series of 10/12 samples to verify the correspondence of the chromatographic conditions to both the elution time and quantities calculated from the calibration curves. The overall sensitivity for the compounds ranged from 2 pg/10 μl for DA to 18 pg/10 μl for HVA with a signal/noise ratio of 3:1.

### Statistical Analysis

Data are presented as mean ± s.e.m. Linear regression, one or two-way ANOVA, and *post hoc* Newman–Keuls tests were used for statistical comparisons, with a significance level of *p* < 0.05.

## Results

### PT320 Reduces L-DOPA/Benserazide-Induced Abnormal Involuntary Movements

**FIGURE 3:**
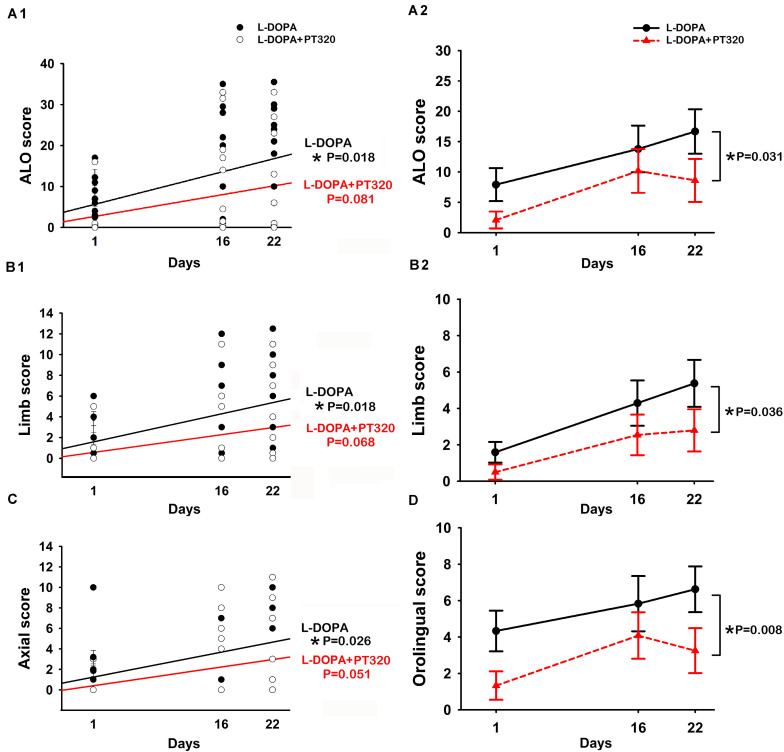
PT320 reduces L-DOPA/benserazide-induced abnormal involuntary movements (AIMs) in unilaterally 6-OHDA-lesioned rats. **(A)** The dyskinesia (**A1**: ALO; **B1**: limbs; and **C:** axial) significantly correlates with the duration of L-DOPA/benserazide treatment. PT320 administration significantly reduced **(A2)** ALO, **(B2)** limb, and **(D)** orolingual AIM scores.

A total of 24 rats received unilateral 6-OHDA lesioning. Of these, 12 rats received daily L-DOPA/benserazide only, and the other 12 rats received daily L-DOPA/benserazide + weekly PT320 for 3 weeks. LID was examined on days 1, 16, and 22 after the initiation of L-DOPA/benserazide treatment (Figure 3). A significant correlation was found between the duration of L-DOPA/benserazide treatment and overall AIM scores(Figure 3A1: ALO, *p* = 0.018, *R* = 0.398). The AIMs on limb (*p* = 0.018, *R* = 0.398) (Figure 3B1) and axial (*p* = 0.026, *R* = 0.370) (Figure 3C) were also significantly correlated with days of L-DOPA/benserazide treatment. In contrast, in the animals receiving PT320, the AIM score was not significantly correlated with the duration of L-DOPA/benserazide treatment (Figure 3A1, ALO, *p* = 0.081; B1: limb, *p* = 0.068; C: axial, *p* = 0.051). These data suggest that L-DOPA/benserazide treatment time-dependently increased dyskinesia, which was attenuated by PT320.

Next, the interaction of PT320 and AIMs was analyzed by a two-way ANOVA with a Newman–Keuls *post hoc* test. The ALO was significantly reduced by PT320 [*p* = 0.031, *F*_(__1, 66__)_ = 4.858] (Figure 3A2). PT320 also significantly reduced limb [*p* = 0.036, *F*_(__1, 64__)_ = 4.582] (Figure 3B2) and orolingual AIM scores [*p* = 0.008, *F*_(__1, 66__)_ = 7.418] (Figure 3D).

### PT320 Normalized DA Turnover in the Lesioned Striatum

**FIGURE 4:**
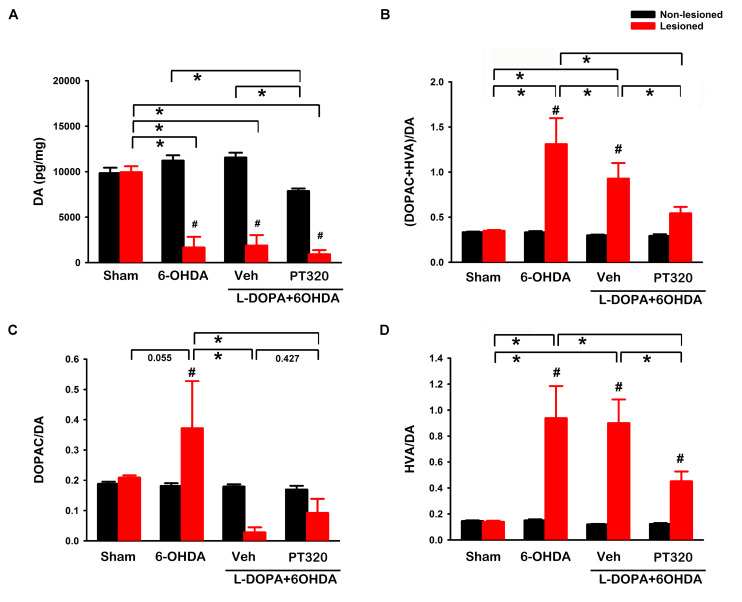
Dopamine (DA) and DA turnover in the striatum. **(A)** 6-OHDA lesioning significantly reduced DA levels (*p < 0.001) in the lesioned striatum (red bars). L-DOPA/benserazide or L-DOPA/benserazide + PT320 treatment did not alter DA after lesioning (*p* = 0.844, 6-OHDA vs. L-DOPA/benserazide + 6-OHDA; *p* = 0.491, 6-OHDA vs. L-DOPA/benserazide + PT320 + 6-OHDA). **(B)** DA turnover was examined by comparing DA metabolite levels (DOPAC + HVA) with DA. DA turnover was enhanced by the 6-OHDA lesion (*p < 0.001, sham vs. 6-OHDA). PT320 significantly reduced DA turnover in the lesioned striatum (*p = 0.028, 6-OHDA + L-DOPA/benserazide vs. 6-OHDA + L-DOPA/benserazide + PT320). 6-OHDA lesioning significantly increased **(C)** DOPAC/DA (^#^*p* = 0.019, lesioned vs. non-lesioned striatum) and **(D)** HVA/DA ratio (^#^*p* < 0.001, lesioned vs. non-lesioned striatum; *p < 0.001, sham vs. 6-OHDA). HVA/DA ratio was significantly reduced by PT320 in the lesioned animals receiving L-DOPA (*p = 0.006, 6-OHDA + L-DOPA/benserazide vs. 6-OHDA + L-DOPA/benserazide + PT320). ^#^Significant difference between the lesioned and non-lesioned side striatum; *significant difference among groups. Two-way ANOVA + *post hoc* Newman–Keuls test.

Lesioned and non-lesioned side striata were collected from 46 rats for HPLC analysis (sham, *n* = 10; lesioned, *n* = 12; lesioned + L-DOPA/benserazide, *n* = 12; and lesioned + L-DOPA/benserazide + PT320, *n* = 12). An averaged 84.6 ± 5.7% reduction of DA was found in the lesioned striatum (*n* = 36). DA levels were significantly reduced in the lesioned side striatum, compared to the non-lesioned side striatum of rats receiving L-DOPA/benserazide (*p* < 0.001) (Figure 4A), L-DOPA/benserazide + PT320 treatment (*p* < 0.001), or without L-DOPA/benserazide treatment (*p* < 0.001). In contrast, no difference was found in the control animals receiving sham surgery (*p* = 0.930). DA levels on the lesioned (right) side striatum of all 6-OHDA-lesioned animals were further analyzed by a two-way ANOVA. L-DOPA/benserazide or L-DOPA/benserazide + PT320 treatment did not alter DA levels in the lesioned striatum (L-DOPA/benserazide + 6-OHDA vs. 6-OHDA: *p* = 0.844; L-DOPA/benserazide + PT320 + 6-OHDA vs. 6-OHDA, *p* = 0.491; L-DOPA/benserazide + PT320 + 6-OHDA vs. L-DOPA/benserazide + 6-OHDA, *p* = 0.649) (Figure 4A). DOPAC and HVA levels were also significantly reduced on the lesioned side striatum after 6-OHDA lesioning (*p* < 0.001). PT320 or L-DOPA/benserazide treatment did not significantly alter DOPAC or HVA levels in the lesioned side striatum (*p* > 0.399).

DA turnover was examined by comparing 3,4-dihydroxyphenylacetic acid (DOPAC) + homovanillic acid (HVA) with DA (Figure 4B). 6-OHDA lesioning significantly increased DA turnover in the striatum (sham vs. 6-OHDA, *p* < 0.001). A similar response was found in animals receiving L-DOPA/benserazide (sham vs. 6-OHDA + L-DOPA/benserazide, *p* = 0.004). Importantly, PT320 significantly reduced DA turnover in the lesioned striatum (*p* = 0.028, 6-OHDA + L-DOPA/benserazide vs. 6-OHDA + L-DOPA/benserazide + PT320) (Figure 4B). 6-OHDA lesioning significantly increased DOPAC/DA (*p* = 0.019, lesioned vs. non-lesioned striatum) (Figure 4C) as well as the HVA/DA ratio (*p* < 0.001, lesioned vs. non-lesioned striatum; *p* < 0.001, sham vs. 6-OHDA) (Figure 4D). Similar to the DA turnover noted above, the HVA/DA ratio was significantly reduced by PT320 in the lesioned striatum (^∗^*p* = 0.006, 6-OHDA + L-DOPA/benserazide vs. 6-OHDA + L-DOPA/benserazide + PT320).

### Interaction of AIM and DA Turnover

All lesioned animals receiving L-DOPA/benserazide (with or without PT320) were pooled for the correlation analysis. ALO score on day 22 was significantly correlated with lesioned side DA turnover [ALO = 3.384 + (12.465 ^∗^ DA turnover), *p* = 0.023, *R* = 0.471, *n* = 23].

## Discussion

LID was examined in unilaterally 6-OHDA−lesioned rats during a 22-day L-DOPA/benserazide treatment. L-DOPA/benserazide or PT320 was administered to animals with an 84% reduction in DA. We found that L-DOPA/benserazide increased AIMs on limbs and axial parameters as well as ALO, the sum of all dyskinesia scores, over 3 weeks. Treatment with PT320 reduced the ALO AIM score and normalized DA turnover. The main finding of this study is that PT320 attenuates LID in PD-like animals.

PT320 is a new SR formulation that provides controlled, continuous release of clinical-grade exendin-4 following s.c. administration across mice (Bader et al., 2019), rats (Chen et al., 2018), non-human primates (Li et al., 2019), and humans (Gu et al., 2014). In this regard, a regulated, initial rapid-release burst provides therapeutic levels of drug in plasma within a few hours, as exendin-4 is liberated from the surface of the injected PLGA microspheres. This is followed by slower secondary and tertiary release phases associated with microsphere hydration that creates an *in situ* matrix drug reservoir from which hydrolysis and erosion of the PLGA polymer subsequently occurs, and results in steady-state exendin-4 release and the long-term maintenance of therapeutic drug levels (Schwendeman et al., 2014; Wan and Yang, 2016).

The continuous release of exendin-4 from SR formulations, such as PT320, as a mechanism to maintain therapeutic drug levels, differs from long-acting GLP-1R agonists that are either covalently linked or bind to large proteins, such as the Fc fragment of human IgG4 (dulaglutide) or human albumin (albiglutide and semiglutide) to reduce clearance and, thereby, maintain GLP-1R agonist levels in plasma. Whereas the brain uptake of Exendin-4 has been reported as approximately 1–2% of its concomitant plasma level across rodent and human studies (Athauda et al., 2017; Chen et al., 2018; Bader et al., 2019; Mullins et al., 2019), that of protein-linked GLP-1R agonists remains unknown but is likely exceedingly low (Kim et al., 2010). Notably, PT320 is currently in Phase IIa clinical trials to evaluate its efficacy and safety in patients with early Parkinson’s disease (ClinicalTrials.gov Identifier: NCT04269642), as exendin-4 provided via PT320 s.c. administration resulted in substantially greater brain penetration than twice-daily administration of immediate-release exendin-4 (Chen et al., 2018; Bader et al., 2019).

We previously reported that PT320, given before or 6 days after 6-OHDA lesioning, significantly improved TH-IR in the lesioned striatum and SNc, and attenuated methamphetamine-induced rotation (Chen et al., 2018). In this study, PT320 was given at 4 weeks after 6-OHDA lesioning. Using HPLC analysis, we found that delayed PT320 treatment did not alter DA levels in the lesioned striatum when the lesion was close to complete. These data suggest that while PT320 reduces the progression of DA degeneration, the protective response to PT320 requires early treatment in this PD model and, on translating this to humans, should best be initiated during the early disease course.

In contrast to its protective effect against DA degeneration in the early stages of PD (Chen et al., 2018), PT320 reduces L-DOPA/benserazide-induced AIMs at 7 weeks after lesioning. Chronic L-DOPA/benserazide treatment for 3 weeks significantly increased AIM (LID). PT320 significantly reduced the AIM score and its correlation with L-DOPA/benserazide treatment in these 6-OHDA rats. We found that the increase in ALO scores significantly correlated with reduced DA turnover, but not DA or its metabolites in the lesioned striatum. Similar findings have been reported in that PD patients with dyskinesia had higher DA turnover in the putamen (Lohle et al., 2016) and HVA/DA in cerebrospinal fluid (Lunardi et al., 2009) than those without dyskinesia. Associated with the reduction of the AIM score, animals receiving PT320 here also had lower DA turnover. Taken together, these data support the interaction of AIM and DA turnover. DA turnover, but not the levels of DA, HVA, or DOPAC, may be a good biomarker for AIM. The mechanisms underlying DA turnover and LID, however, require further investigation.

Besides LID, DA graft-induced dyskinesia (GID) has been reported in selective PD cases (Ma et al., 2002) and in animals receiving amphetamine injection (Smith et al., 2012). However, GID was not found in MPTP-treated monkeys receiving intraputamenal grafts of fetal dopaminergic cells (Kordower et al., 2017). Furthermore, embryonic dopamine neuronal grafts improved L-DOPA-mediated AIM and normalized preproenkephalin and prodynorphin expression in the indirect and direct pathway in 6-OHDA-lesioned rats (Lee et al., 2000). These data suggest that differential mechanisms of dyskinesia may be involved in LID and GID. The use of PT320 in preventing GID requires further investigation. In the light of the positive actions of PT320 on LID in the current study, the evaluation of PT320 in preventing GID warrants investigation as our understanding and present treatment options of GID are strictly limited (Tronci et al., 2015; Lane, 2019).

PT320 significantly reduced DA turnover in the 6-OHDA-lesioned striatum. On the other hand, PT320 or L-DOPA/benserazide did not alter DA, DOPAC, or HVA levels in the lesioned striatum. The “normalization” of DA turnover in the 6-OHDA lesioned animals treated with PT320 suggests increased conversion of extracellular DA to DA metabolites in these animals. The presynaptic terminals of nigrostriatal DA fibers have D2 autoreceptors whose stimulation by extracellular DA inhibits DA synthesis and release, and subsequent metabolism to HVA and DOPAC. The reduction in turnover implies more extracellular DA availability in response to activation of these inhibitory D2 autoreceptors by DA after PT320 treatment. This would be readily reflected in DA turnover or the HVA/DA ratio, but not in DA levels.

## Conclusion

In conclusion, our data support the notion that PT320 reduced the L-DOPA/benserazide−mediated AIM in a 6-OHDA rat model of PD, likely through the modulation of DA turnover in the lesioned brain. Our studies further emphasize the potential utility of PT320 in the treatment of clinical PD, for which clinical trials are ongoing, and highlight the opportunity to mitigate LID, a common and disabling feature of L-DOPA treatment that can reduce its beneficial effects.

## Data Availability Statement

All datasets presented in this study are included in the article/supplementary material.

## Ethics Statement

The animal study was reviewed and approved by the National Health Research Institutes.

## Author Contributions

S-JY, SC, and Y-YY analyzed the data. EG, JJ, HK, H-IC, and D-SC developed the dosing, provided the materials, and performed the experiments. BH, NG, H-IC, and D-SC conceptualized the study. YW and S-JY wrote the initial draft. All authors edited the manuscript.

## Conflict of Interest

JJ, S-JY, and H-IC are employees of Peptron Inc. D-SC is a scientific advisor to Peptron Inc. The Intramural Research Program of the National Institute on Aging, N.I.H., and Peptron Inc. have a Cooperative Research and Development Agreement to develop Exendin-4 as a treatment strategy for neurodegenerative disorders for which NIA and Peptron Inc. hold patent rights via the work of H-IC and NG. The remaining authors declare that the research was conducted in the absence of any commercial or financial relationships that could be construed as a potential conflict of interest. The handling Editor is currently organizing a Research Topic with one of the authors, NG.

## Abbreviations

6-OHDA: 6-hydroxydopamine
AIMs: abnormal involuntary movements
ALO: sum of all AIMs (axial limb and oro-lingual) score
DA: dopamine
DOPAC: 3,4-dihydroxyphenylacetic acid
GID: graft -induced dyskinesia
GLP-1: glucagon-like peptide-1
GLP-1R: GLP-1 receptor
GPe: external segment of globus pallidus
GPi: internal segment of globus
HVA: homovanillic acid
L-DOPA: L-3,4-dihydroxyphenylalanine
LID: levodopa-induced dyskinesia
MPTP: 1-methyl-4-phenyl-1,2,3,6-tetrahydropyridine
MSNs: medium spiny neurons
PD: Parkinson’s disease
s.c.: subcutaneous
SR: sustained-release
STN: subthalamic nucleus
T2DM: type 2 diabetes mellitus
TH-IR: tyrosine hydroxylase immunoreactivity.

## References

[B1] Abuirmeileh A. Harkavyi A. Rampersaud N. Lever R. Tadross J. A. Bloom S. R. (2012). Exendin-4 treatment enhancesL-Dopa evoked release of striatal dopamine and decreases dyskinetic movements in the 6-hydoxydopamine lesioned rat. *J. Pharm. Pharmacol.* 64 637–643. 22471359 10.1111/j.2042-7158.2011.01394.x

[B2] Athauda D. Foltynie T. (2018). Protective effects of the GLP-1 mimetic exendin-4 in Parkinson’s disease. *Neuropharmacology* 136 260–270. 10.1016/j.neuropharm.2017.09.023 28927992

[B3] Athauda D. Gulyani S. Karnati H. K. Li Y. Tweedie D. Mustapic M. (2019). Utility of neuronal-derived exosomes to examine molecular mechanisms that affect motor function in patients with parkinson disease: a secondary analysis of the exenatide-PD Trial. *JAMA Neurol.* 76 420–429. 30640362 10.1001/jamaneurol.2018.4304 PMC6459135

[B4] Athauda D. Maclagan K. Skene S. S. Bajwa-Joseph M. Letchford D. Chowdhury K. (2017). Exenatide once weekly versus placebo in Parkinson’s disease: a randomised, double-blind, placebo-controlled trial. *Lancet* 390 1664–1675. 28781108 10.1016/S0140-6736(17)31585-4 PMC5831666

[B5] Bader M. Li Y. Lecca D. Rubovitch V. Tweedie D. Glotfelty E. (2019). Pharmacokinetics and efficacy of PT302, a sustained-release Exenatide formulation, in a murine model of mild traumatic brain injury. *Neurobiol. Dis.* 124 439–453. 10.1016/j.nbd.2018.11.023 30471415 PMC6710831

[B6] Baron M. S. Vitek J. L. Bakay R. A. Green J. McDonald W. M. Cole S. A. (2000). Treatment of advanced Parkinson’s disease by unilateral posterior GPi pallidotomy: 4-year results of a pilot study. *Mov. Disord.* 15 230–237. 10.1002/1531-8257(200003)15:2<230::aid-mds1005>3.0.co;2-u 10752571

[B7] Bergman H. Wichmann T. DeLong M. R. (1990). Reversal of experimental parkinsonism by lesions of the subthalamic nucleus. *Science* 249 1436–1438. 10.1126/science.2402638 2402638

[B8] Bjornestad A. Forsaa E. B. Pedersen K. F. Tysnes O. B. Larsen J. P. Alves G. (2016). Risk and course of motor complications in a population-based incident Parkinson’s disease cohort. *Parkinsonism. Relat. Disord.* 22 48–53. 10.1016/j.parkreldis.2015.11.007 26585090

[B9] Chen S. Yu S. J. Li Y. Lecca D. Glotfelty E. Kim H. K. (2018). Post-treatment with PT302, a long-acting Exendin-4 sustained release formulation, reduces dopaminergic neurodegeneration in a 6-hydroxydopamine rat model of Parkinson’s disease. *Sci. Rep.* 8:10722. 10.1038/s41598-018-28449-z PMC6048117 30013201

[B10] Drucker D. J. (2018). Mechanisms of Action and Therapeutic Application of Glucagon-like Peptide-1. *Cell Metab.* 27 740–756. 10.1016/j.cmet.2018.03.001 29617641

[B11] Durieux P. F. Schiffmann S. N. de Kerchove D. A. (2011). Targeting neuronal populations of the striatum. *Front. Neuroanat* 5:40. 10.3389/fnana.2011.00040 21811438 PMC3139926

[B12] Espay A. J. Morgante F. Merola A. Fasano A. Marsili L. Fox S. H. (2018). Levodopa-induced dyskinesia in Parkinson disease: current and evolving concepts. *Ann. Neurol.* 84 797–811. 10.1002/ana.25364 30357892

[B13] Fox S. H. Brotchie J. M. Lang A. E. (2008). Non-dopaminergic treatments in development for Parkinson’s disease. *Lancet Neurol.* 7 927–938. 10.1016/s1474-4422(08)70214-x 18848312

[B14] Gentilella R. Pechtner V. Corcos A. Consoli A. (2019). Glucagon-like peptide-1 receptor agonists in type 2 diabetes treatment: are they all the same? *Diabetes Metab. Res. Rev.* 35:e3070. 10.1002/dmrr.3070 30156747

[B15] Gerfen C. R. Surmeier D. J. (2011). Modulation of striatal projection systems by dopamine. *Annu. Rev. Neurosci.* 34 441–466. 10.1146/annurev-neuro-061010-113641 21469956 PMC3487690

[B16] Glotfelty E. J. Delgado T. Tovar Y. R. L. Luo Y. Hoffer B. Olson L. (2019). Incretin mimetics as rational candidates for the treatment of traumatic brain injury. *ACS Pharmacol. Transl. Sci.* 2 66–91. 10.1021/acsptsci.9b00003 31396586 PMC6687335

[B17] Glotfelty E. J. Olson L. Karlsson T. E. Li Y. Greig N. H. (2020). Glucagon-like peptide-1 (GLP-1)-based receptor agonists as a treatment for Parkinson’s disease. *Expert Opin. Investig. Drugs* 10.1080/13543784.2020.1764534 [Online ahead of print], 32412796 PMC10477949

[B18] Gu N. Cho S. H. Kim J. Shin D. Seol E. Lee H. (2014). Pharmacokinetic properties and effects of PT302 after repeated oral glucose loading tests in a dose-escalating study. *Clin. Ther.* 36 101–114. 10.1016/j.clinthera.2013.12.002 24373998

[B19] Holscher C. (2020). Brain insulin resistance: role in neurodegenerative disease and potential for targeting. *Expert. Opin. Investig. Drugs* 29 333–348. 10.1080/13543784.2020.1738383 32175781

[B20] Jenner P. (2008). Molecular mechanisms of L-DOPA-induced dyskinesia. *Nat. Rev. Neurosci.* 9 665–677. 10.1038/nrn2471 18714325

[B21] Kim B. J. Zhou J. Martin B. Carlson O. D. Maudsley S. Greig N. H. (2010). Transferrin fusion technology: a novel approach to prolonging biological half-life of insulinotropic peptides. *J. Pharmacol. Exp. Ther.* 334 682–692. 10.1124/jpet.110.166470 20498254 PMC2939671

[B22] Kim D. S. Choi H. I. Wang Y. Luo Y. Hoffer B. J. Greig N. H. (2017). A new treatment strategy for Parkinson’s disease through the gut-brain axis: the Glucagon-like Peptide-1 receptor pathway. *Cell Transplant.* 26 1560–1571. 10.1177/0963689717721234 29113464 PMC5680957

[B23] Kordower J. H. Vinuela A. Chu Y. Isacson O. (2017). Parkinsonian monkeys with prior levodopa-induced dyskinesias followed by fetal dopamine precursor grafts do not display graft-induced dyskinesias. *J. Comp. Neurol.* 525 498–512. 10.1002/cne.24081 27418401

[B24] Lane E. L. (2019). L-DOPA for Parkinson’s disease-a bittersweet pill. *Eur. J. Neurosci.* 49 384–398. 10.1111/ejn.14119 30118169

[B25] Lee C. S. Cenci M. A. Schulzer M. Bjorklund A. (2000). Embryonic ventral mesencephalic grafts improve levodopa-induced dyskinesia in a rat model of Parkinson’s disease. *Brain* 123 (Pt 7), 1365–1379. 10.1093/brain/123.7.1365 10869049

[B26] Li Y. Perry T. Kindy M. S. Harvey B. K. Tweedie D. Holloway H. W. (2009). GLP-1 receptor stimulation preserves primary cortical and dopaminergic neurons in cellular and rodent models of stroke and Parkinsonism. *Proc. Natl. Acad. Sci U.S.A.* 106 1285–1290. 10.1073/pnas.0806720106 19164583 PMC2633544

[B27] Li Y. Vaughan K. L. Tweedie D. Jung J. Kim H. K. Choi H. I. (2019). Pharmacokinetics of Exenatide in nonhuman primates following its administration in the form of sustained-release PT320 and Bydureon. *Sci. Rep.* 9:17208. 10.1038/s41598-019-53356-2 PMC6868133 31748513

[B28] Lohle M. Mende J. Wolz M. Beuthien-Baumann B. Oehme L. van den Hoff J. (2016). Putaminal dopamine turnover in de novo Parkinson disease predicts later motor complications. *Neurology* 86 231–240. 10.1212/wnl.0000000000002286 26718573

[B29] Lunardi G. Galati S. Tropepi D. Moschella V. Brusa L. Pierantozzi M. (2009). Correlation between changes in CSF dopamine turnover and development of dyskinesia in Parkinson’s disease. *Parkinsonism. Relat. Disord.* 15 383–389. 10.1016/j.parkreldis.2008.10.001 19010710

[B30] Lundblad M. Andersson M. Winkler C. Kirik D. Wierup N. Cenci M. A. (2002). Pharmacological validation of behavioural measures of akinesia and dyskinesia in a rat model of Parkinson’s disease. *Eur. J. Neurosci.* 15 120–132. 10.1046/j.0953-816x.2001.01843.x 11860512

[B31] Ma Y. Feigin A. Dhawan V. Fukuda M. Shi Q. Greene P. (2002). Dyskinesia after fetal cell transplantation for parkinsonism: a PET study. *Ann. Neurol.* 52 628–634. 10.1002/ana.10359 12402261

[B32] Manson A. Stirpe P. Schrag A. (2012). Levodopa-induced-dyskinesias clinical features, incidence, risk factors, management and impact on quality of life. *J. Parkinsons Dis.* 2 189–198. 10.3233/jpd-2012-120103 23938226

[B33] Merello M. Balej J. Delfino M. Cammarota A. Betti O. Leiguarda R. (1999). Apomorphine induces changes in GPi spontaneous outflow in patients with Parkinson’s disease. *Mov. Disord.* 14 45–49. 10.1002/1531-8257(199901)14:1<45::aid-mds1009>3.0.co;2-f 9918343

[B34] Mullins R. J. Mustapic M. Chia C. W. Carlson O. Gulyani S. Tran J. (2019). A pilot study of Exenatide actions in Alzheimer’s disease. *Curr. Alzheimer Res.* 16 741–752. 10.2174/1567205016666190913155950 31518224 PMC7476877

[B35] Nambu A. Tokuno H. Takada M. (2002). Functional significance of the cortico-subthalamo-pallidal ‘hyperdirect’ pathway. *Neurosci. Res.* 43 111–117. 10.1016/s0168-0102(02)00027-5 12067746

[B36] Nicoletti A. Mostile G. Nicoletti G. Arabia G. Iliceto G. Lamberti P. (2016). Clinical phenotype and risk of levodopa-induced dyskinesia in Parkinson’s disease. *J. Neurol.* 263 888–894. 26964541 10.1007/s00415-016-8075-6

[B37] Nutt J. G. Chung K. A. Holford N. H. (2010). Dyskinesia and the antiparkinsonian response always temporally coincide: a retrospective study. *Neurology* 74 1191–1197. 10.1212/wnl.0b013e3181d90050 20220120 PMC2865731

[B38] Papa S. M. Desimone R. Fiorani M. Oldfield E. H. (1999). Internal globus pallidus discharge is nearly suppressed during levodopa-induced dyskinesias. *Ann. Neurol.* 46 732–738. 10.1002/1531-8249(199911)46:5<732::aid-ana8>3.0.co;2-q 10553990

[B39] Peptron Inc (2020). *SmartDepotTM technology.* Available online at: http://peptron.com/ds2_2_1.html (accessed April 1, 2020).

[B40] Petri D. Pum M. Vesper J. Huston J. P. Schnitzler A. (2013). GABAA-receptor activation in the subthalamic nucleus compensates behavioral asymmetries in the hemiparkinsonian rat. *Behav. Brain Res.* 252 58–67. 10.1016/j.bbr.2013.05.044 23727148

[B41] Putterman D. B. Munhall A. C. Kozell L. B. Belknap J. K. Johnson S. W. (2007). Evaluation of levodopa dose and magnitude of dopamine depletion as risk factors for levodopa-induced dyskinesia in a rat model of Parkinson’s disease. *J. Pharmacol. Exp. Ther.* 323 277–284. 10.1124/jpet.107.126219 17660384

[B42] Rascol O. Brooks D. J. Korczyn A. D. De Deyn P. P. Clarke C. E. Lang A. E. (2000). A five-year study of the incidence of dyskinesia in patients with early Parkinson’s disease who were treated with ropinirole or levodopa. *N. Engl. J Med.* 342 1484–1491. 10.1056/nejm200005183422004 10816186

[B43] Schwendeman S. P. Shah R. B. Bailey B. A. Schwendeman A. S. (2014). Injectable controlled release depots for large molecules. *J. Control Release* 190 240–253. 10.1016/j.jconrel.2014.05.057 24929039 PMC4261190

[B44] Smith G. A. Heuer A. Klein A. Vinh N. N. Dunnett S. B. Lane E. L. (2012). Amphetamine-induced dyskinesia in the transplanted hemi-Parkinsonian mouse. *J. Parkinsons Dis.* 2 107–113. 10.3233/jpd-2012-12102 23933747

[B45] Touchon J. C. Moore C. Frederickson J. Meshul C. K. (2004). Lesion of subthalamic or motor thalamic nucleus in 6-hydroxydopamine-treated rats: effects on striatal glutamate and apomorphine-induced contralateral rotations. *Synapse* 51 287–298. 10.1002/syn.10306 14696016

[B46] Tronci E. Fidalgo C. Carta M. (2015). Fetal cell transplantation for Parkinson’s disease: focus on graft-induced dyskinesia. *Parkinsons Dis*. 2015:563820. 10.1155/2015/563820 26881178 PMC4736211

[B47] Wan F. Yang M. (2016). Design of PLGA-based depot delivery systems for biopharmaceuticals prepared by spray drying. *Int. J. Pharm.* 498 82–95. 10.1016/j.ijpharm.2015.12.025 26688034

